# Machine says go, doctor says no: an ecological momentary assessment analysis examining clinicians’ perceptions of, and their antibiotic prescribing behaviour when using rapid molecular diagnostic tests in intensive care

**DOI:** 10.1186/s13756-025-01690-8

**Published:** 2026-03-24

**Authors:** Sarah-Jane F. Stewart, Virve I. Enne, Alyssa M. Pandolfo, Yogini H. Jani, David Brealey, Stephen J. Brett, David M. Livermore, Vanya Gant, Rob Horne, the INHALE WP Study Group, Julie A. Barber, Zaneeta Dhesi, Zoe Moon, Mark Peters, Juliet High, Charlotte Russell, Susan Stirling, Antony Colles, Kerry Dresser, Ann Marie Swart, David Turner, Adam Wagner, Valerie Page, Hala Kandil, Ingeborg Welters, Robert Parker, Mark de Neef, Damien Mack, Q Emmanuel, Nehal Patel, Suveer Singh, Luke Moore, Nabeela Mughal, Jane Cassidy

**Affiliations:** 1 UCL School of Pharmacy, University College London, London, UK; 2 School of Psychology, University of Surrey, Guildford, UK; 3 Division of Infection and Immunity, Faculty of Medical Sciences, University College London, London, UK; 4 Centre for Medicines Optimisation Research and Education, University College London Hospitals NHS Foundation Trust, London, UK; 5 Division of Critical Care, University College London Hospitals NHS Foundation Trust, London, UK; 6 Department of Surgery and Cancer, Imperial College London, London, UK; 7 Norwich Medical School, University of East Anglia, Norwich, UK; 8 Department of Medical Microbiology, University College London Hospitals NHS Foundation Trust, London, UK

**Keywords:** Rapid molecular diagnostics, Antibiotic stewardship, Prescribing behaviour, Prescribing decision-making

## Abstract

**Background:**

Rapid molecular diagnostics such as the BioFire FilmArray Pneumonia Panel (the Pneumonia Panel) can improve antibiotic stewardship by supporting doctors to make more targeted antibiotic prescribing decisions faster compared to routine microbiology. However, factors influencing how these test results translate to individual prescribing decisions are poorly understood. The INHALE randomised controlled trial (RCT) evaluated the application of the Pneumonia Panel to manage suspected hospital-acquired and ventilator-associated pneumonias (HAP/VAP) in English intensive care unit (ICU) patients. This behavioural study examines clinicians perceived and actual antibiotic prescribing behaviour, within the INHALE RCT.

**Methods:**

Clinicians treating ICU patients completed brief questionnaires within 24 h of their prescribing decision for intervention-arm cases (N = 159), exploring factors influencing their decision and perceptions about the test results. Actual prescribing behaviour was extracted from the trial database. A 4-block hierarchical logistic regression identified predictors of prescriptions being consistent with Pneumonia Panel results.

**Results:**

65% (N = 104) of prescribing decisions were consistent with Pneumonia Panel results. The test result itself was a dominant factor: 88% (N = 98) of decisions were consistent when results were positive (pathogens found). However, only 13% (N = 6) of decisions were consistent when no pathogens were detected. Consequently, clinicians were often reluctant to eschew initial antibiotics or de-escalate early where appropriate, ‘*erring on the side of caution*’. Clinicians perceptions, specifically the speed of results, concurrent antibiotic treatment, the patient having additional confirmed evidence of infection, and believing the patient is unlikely to have a non-respiratory infection predicted prescribing decisions being aligned with test results (all p < .05).

**Conclusions:**

Findings have implications for the roll-out of rapid diagnostics in practice, particularly regarding the management of negative results. Implementation strategies need to be behaviourally intelligent, connecting with how clinicians think and behave.

**Supplementary Information:**

The online version contains supplementary material available at 10.1186/s13756-025-01690-8.

## Background

Early well-targeted antibiotics reduce mortality in hospital-acquired and ventilator-associated pneumonias (HAP/VAP). However standard microbiological investigation takes circa 48–72 h to produce results needed to guide treatment [1]. In the interim antibiotic therapy is empirical and usually broad spectrum. Rapid molecular diagnostic tests such as the FilmArray [2] and Unyvero [3] can detect multiple respiratory pathogen and resistance genes directly from respiratory secretions with fast (1-6 h) results, delivered at the point-of-care[4]. This may improve stewardship by swiftly guiding narrower-spectrum prescriptions [5, 6].

Although clinicians value the speed of these tests, many express concerns about their clinical application (e.g. consequences of under-prescribing) [7–10]. Conventional decisions to prescribe a broad-spectrum empirical antibiotic are often driven by an immediate desire to protect the patient and prescriber, ‘*erring on the side of caution’.* Concerns about generating antimicrobial resistance (AMR), exacerbated by over-prescription of broad-spectrum agents, are more distal [7]

Our previous work has identified key factors important to antimicrobial prescribing decisions in HAP/VAP [7, 8, 10]. However, we have little insight into how these competing factors contribute in practice. Particularly, which factors are most influential in determining a clinicians’ willingness to apply results of rapid diagnostics to individual prescribing decisions where there is considerable uncertainty.

Much of our previous work has been qualitative [7, 8, 10], exploring clinicians’ perceptions about antibiotic prescribing and the use of rapid diagnostics in-depth. However, this relied on clinicians’ recall and ability to provide a summary view over a range of prescribing instances. In contrast, the present investigation examines clinicians’ perceptions of rapid diagnostic results, and their actual antibiotic prescribing behaviour, within the context of the INHALE randomised controlled trial (RCT). This was a large multi-centre study, comparing stewardship and outcomes for HAP/VAP patients whose treatment was either guided by (i) local guidelines until routine microbiology results became available, or (ii) the BioFire Pneumonia Panel and an optional prescribing algorithm [11, 12]. Specifically, here we examined i) what proportion of ICU antibiotic prescribing decisions were consistent with the Pneumonia Panel test results? and ii) what factors predicted prescribing decisions being consistent with Pneumonia Panel test results?

## Methods

This study is part of the INHALE research programme (ISRCTN16483855), funded by the UK (United Kingdom) National Institute for Health Research. INHALE investigated the utility of molecular diagnostics to guide antimicrobial prescribing for ICU patients with suspected HAP/VAP. The INHALE RCT randomised patients with suspected HAP/VAP at 14 ICUs to i) standard empirical antibiotics, adapted once routine microbiology results became available, or ii) initial care informed by the Pneumonia Panel [12] with an optional prescribing algorithm translating results into antibiotic therapy suggestions [11], adapted once routine microbiology results became available. The Pneumonia Panel uses multiplex polymerase-chain reaction technology to seek pathogens and resistance genes (Supplementary Material 1). The INHALE trial prescribing algorithm was adapted to accommodate appropriate site-specific requirements and where possible, recommended narrow-spectrum antibiotics, a copy of which can be found here:[11]. Further information relating to sample types and additional RCT method details can be found here:[12]. An observational, behavioural evaluation of ICU clinicians’ antibiotic prescribing was embedded within the RCT and is reported here. A sample of clinicians responsible for treating INHALE RCT patients completed brief questionnaires within 24 h of arm-randomisation and prescribing decision.

### Sample and setting

This study took place in 13 of the 14 English ICUs^1^ participating in the INHALE RCT (Supplementary Material 2 for hospital and ICU characteristics). Clinical staff making antibiotic decisions for RCT patients were eligible.

Given the novel nature of this research, a formal power calculation was not conducted due to a lack of data on expected clinician behaviour and perceptions using the present study design. However, we applied the widely used criterion of 10 events-per-variable (EVP) [13], and minimum of 100 events to be sufficient for a predictive logistic regression model [14], when sampling for this study.

### Data collection

Questionnaires were administered in ICUs between October 2019 and July 2021. Clinicians primarily responsible for treating INHALE RCT patients were invited to complete one questionnaire per patient but could complete questionnaires for multiple patients. Questionnaires were completed after clinicians made an antibiotic decision following receiving Pneumonia Panel results (Supplementary Material 3)^2^ After 24 h, incomplete questionnaires were classified as non-responses, and disregarded, to minimise recall bias. The study questionnaire is available from the authors upon request.

### Measures

#### Primary outcome variable:

##### Consistency of antibiotic prescribing decisions with Pneumonia Panel results

Decisions were scored as consistent with the Pneumonia Panel result if the test identified at least one pathogen and if antimicrobial(s) with activity^3^ against that pathogen(s) were prescribed irrespective of proportionality (treatment did not entail using an unnecessarily broad-spectrum agent for the pathogen(s) found), or no pathogen(s) were identified, and no antimicrobial was prescribed. Decisions were scored as not consistent with Pneumonia Panel results if the test found at least one pathogen but no antimicrobial(s) with activity against that pathogen were prescribed, or if the test did not identify pathogen(s), but antimicrobial(s) were prescribed.

#### Secondary outcome variables:

##### Pneumonia Panel test result (positive/negative)

Pneumonia Panel test results were coded ‘negative’ if no pathogen(s) were identified. Test results were coded ‘positive’ if at least one pathogen was identified.

##### Perceptions of the Pneumonia Panel test and the patient

Other secondary outcome variables were measured via an ecological momentary assessment (EMA)[15] questionnaire to capture ‘in-the-moment’ factors influencing antibiotic decision-making, to understand prescribing decisions as they happen, and to reduce recall bias. Questionnaire items were developed using our previous work, other published questionnaires and interview schedules, and feedback from clinicians [7, 8, 16–20]. The questionnaire took approximately one minute to complete and recorded basic descriptive information and factors influencing prescribing decisions.

Clinicians rated agreement on a 3-point scale (disagree, uncertain, agree) with the following statements: i) I believed the Pneumonia Panel results, ii) I found the Pneumonia Panel results easy to interpret and understand, iii) the patient was clinically deteriorating, iv) the patient had laboratory or radiological evidence of deterioration, v) the patient likely had other sources of infection besides the lung, and vi) initial antibiotic prescription for respiratory tract infection (RTI) or other infection was appropriate and did not need to be changed. Clinicians ticked a box to indicate if the rapidity of the Pneumonia Panel results influenced their decision. Clinicians could also select ‘not applicable’.

Clinicians ticked boxes to indicate the following local contextual factors that influenced their prescribing decision: i) trust guidelines/Unit standard operating procedures (SoPs), ii) availability of a respiratory culture result prior to recruitment to the study, iii) availability of culture results for another body site, iv) following the local SOS plan (out-of-hours prescribing contingency plan), and v) preserving the ecology of the unit. There was a free-text box for clinicians to indicate other information not captured by the questionnaire.

##### Consistency of antibiotic prescribing decisions with INHALE trial prescribing algorithm

Clinicians indicated on the questionnaire whether they perceived their prescribing decisions to be (in)consistent with the INHALE trial prescribing algorithm recommendations[11].

Clinician’s adherence to the algorithm was objectively assessed by examining whether their actual prescribing was (in) consistent with antimicrobial agent(s) recommended for the pathogen(s) by the algorithm.

### Data analysis

Data were analysed using Jamovi Version 2.5 [21]. Only clinicians treating patients in the intervention-arm had access to Pneumonia Panel results, therefore only intervention-arm cases were included in the present analysis. The prescribing algorithm was only relevant for cases where the test produced a positive result, therefore analyses examining consistency with algorithm recommendations only included those cases.

To examine the proportion of prescribing decisions consistent with Pneumonia Panel results and the algorithm, frequency counts and percentages were calculated. Chi-square tests assessed whether significantly more prescribing decisions were consistent with Pneumonia Panel results, and the INHALE trial prescribing algorithm, than not, and the relationship between self-reported and observed adherence to the algorithm.

Variables reflecting clinicians’ perceptions of the Pneumonia Panel test, and the patient were dichotomised (1 = agreement, 0 = not in agreement), with responses of ‘uncertain’ being classified as ‘not in agreement’.

Chi-square tests examined the factors that significantly related to decisions being consistent with Pneumonia Panel results; significant factors (only) were then entered into a hierarchical logistic regression to assess the comparative predictive influence of each factor. Variables were grouped conceptually to determine the model blocks that would be entered into the regression.

Questionnaire free-text box responses were coded independently by two researchers to extract factors relevant to antibiotic decision-making not otherwise captured by the questionnaire. Coding consistency between coders was checked and was high. No codes made an additional contribution to the existing data.

## Results

Questionnaires were issued for a total of 481 eligible prescribing instances; from these, 295 responses were received (61% response rate). Thirteen responses were excluded from the analyses: 3 patients withdrew from the trial and requested data removal; and 10 were removed due to clinician error (4 control arm, 6 intervention-arm). Among the remaining 282 questionnaires, 159 (56%) related to intervention-arm cases and hence were included in the present analyses, with the remainder from the control arm. Table 1 presents an overview of clinician grade/specialty.

**Table 1:** Overview of clinician speciality/grade completing questionnaires for each prescribing case across the INHALE RCT

	Intervention-arm cases (N = 159, 56.38%)
ICU Consultant (n = 71)	107 (67.30%)
ICU Middle-grade Trainee (n = 24)	27 (16.98%)
ICU Early-grade Trainee (n = 1)	1 (0.63%)
Consultant Microbiologist (n = 4)	22 (13.84%)
Nurse Practitioner (n = 1)	1 (0.63%)
Unknown (n = 1)	1 (0.63%)

### The proportion of prescribing decisions consistent with Pneumonia Panel results

65.4% (N = 104) of prescribing decisions were consistent with Pneumonia Panel results (i.e. a prescription of antimicrobial(s) with activity against pathogen(s) identified by the Pneumonia Panel or no antimicrobial(s) if the test was negative), 34.6% (N = 55) of decisions were not (χ^*2*^(1) = 15.10, *p* < 0.001) (Table 2).

**Table 2:** Factors associated with a prescribing decision consistent with Pneumonia Panel results

	Prescription consistent with Pneumonia Panel result		Chi square
	Yes	No	
*Pneumonia Panel test result*			
Positive (i.e. pathogen(s) found)	98 (88.29%)	13 (11.71%)	X^2^(1) = 85.07, *p* < .001
Negative (i.e. no pathogen(s) found)	6 (12.50%)	42 (87.50%)	
*Clinician believed the Pneumonia Panel results*			
Yes	87 (68.50%)	40 (31.50%)	X^2^(1) = 4.55, *p* = .033
No	11 (45.83%)	13 (54.17%)	
*Clinician found the Pneumonia Panel results easy to interpret and understand*			
Yes	95 (64.19%)	53 (35.81%)	X^2^(1) = 1.41, *p* = .236
No	9 (81.82%)	2 (18.18%)	
*Clinicians influenced by rapidity of Pneumonia Panel results*			
Yes	28 (84.85%)	5 (15.15%)	X^2^(1) = 6.96, *p* = .008
No	76 (60.32%)	50 (39.68%)	
*Patient was clinically deteriorating*			
Yes	82 (67.77%)	39 (32.23%)	X^2^(1) = 1.51, *p* = .219
No	21 (56.76%)	16 (43.24%)	
*Patient had laboratory or radiological evidence of deterioration*			
Yes	87 (69.60%)	38 (30.40%)	X^2^(1) = 4.54, *p* = .033
No	17 (50.00%)	17 (50.00%)	
*Perception that patient likely had other sources of infection besides the lung*			
Yes	14 (40.00%)	21 (60.00%)	X^2^(1) = 12.34, *p* < .001
No	88 (72.13%)	34 (27.87%)	
*Initial antibiotic prescription for RTI or other infection was appropriate and did not need to be changed*			
Yes	45 (60.00%)	30 (40.00%)	X^2^(1) = 2.00, *p* = .157
No	58 (70.73%)	24 (29.27%)	
*Ongoing antibiotics for other body site infection*			
Yes	5 (38.46%)	8 (61.54%)	X^2^(1) = 4.54, *p* = .033
No	99 (67.81%)	47 (32.19%)	
*Trust guidelines/Unit SOPs (availability of)*			
Yes	45 (65.22%)	24 (34.78%)	X^2^(1) = 0.00, *p* = .965
No	59 (65.56%)	31 (34.44%)	
*Available recent respiratory culture result*			
Yes	14 (77.78%)	4 (22.22%)	X^2^(1) = 1.37, *p* = .241
No	90 (63.83%)	51 (36.17%)	
*Available culture result(s) from other body site(s)*			
Yes	2 (33.33%)	4 (66.67%)	X^2^(1) = 2.84, *p* = .092
No	102 (66.67%)	51 (33.33%)	
*Following the SOS (contingency out-of-hours) pla*n			
Yes	6 (60.00%)	4 (40.00%)	X^2^(1) = 0.14, *p* = .710
No	98 (65.77%)	51 (34.23%)	
*Preserving the ecology of the unit influenced clinicians’ prescribing decision*			
Yes	5 (55.56%)	4 (44.44%)	X^2^(1) = 0.41, *p* = .522
No	99 (66.00%)	51 (34.00%)	

### The proportion of prescribing decisions consistent with the prescribing algorithm

Clinicians self-reported their prescribing as consistent with algorithm recommendations in 59.6% (N = 62) of cases, compared with 40.4% (N = 42) that were not (χ^*2*^(1) = 3.85, *p* = 0.05). However, when examining clinician’s observed adherence to the algorithm this was much lower; only 32.4% (N = 36) of decisions were consistent, compared to 67.6% (N = 75) that were not (χ^2^(1) = 13.7, *p* < 0.001). Clinicians’ self-reported adherence to the algorithm was significantly higher than their observed adherence (χ^*2*^(1) = 34.2, *p* < 0.001).

### Factors predicting a prescribing decision consistent with Pneumonia Panel results

Chi-square tests examined the association between secondary outcome variables and the proportion of prescribing decisions consistent with Pneumonia Panel results (Table 2). Receiving a positive result, believing the results, being influenced by the rapidity of Pneumonia Panel results, perceiving the patient to likely not have a non-respiratory source of infection, and the patient not already receiving prior ongoing antibiotics for a non-respiratory infection all significantly related to prescribing decisions consistent with Pneumonia Panel results (all p < 0.05). These factors were entered into a 4-block hierarchical logistic regression (Table 3).

**Table 3:** Model coefficients predicting a prescribing decision being consistent with Pneumonia Panel results

				95% Confidence Interval		
	B	SE	ExpB	Lower	Upper	*p*
Block 1						
Intercept	−2.08	0.47	0.13	0.05	0.32	< .001
Positive Pneumonia Panel result	4.03	0.56	56.00	18.71	167.62	< .001
Block 2						
Intercept	−2.10	0.69	0.12	0.03	0.48	.002
Positive Pneumonia Panel result	4.02	0.57	55.65	18.11	171.06	< .001
Believing Pneumonia Panel results	0.03	0.71	1.04	0.26	4.14	.961
Block 3						
Intercept	−2.89	0.87	0.06	0.01	0.31	< .001
Positive Pneumonia Panel result	4.75	0.79	115.30	24.39	544.95	< .001
Believing Pneumonia Panel results	-0.22	0.73	0.81	0.19	3.39	.767
Influenced by quick speed of Pneumonia Panel	2.70	0.92	14.92	2.44	91.40	.003
Block 4						
Intercept	−4.00	1.04	0.02	0.00	0.14	< .001
Positive Pneumonia Panel result	4.93	0.85	138.16	26.21	728.40	< .001
Believing Pneumonia Panel results	0.18	0.74	0.81	0.28	5.13	.813
Influenced by quick speed of Pneumonia Panel	2.50	0.96	12.15	1.85	79.62	.009
Ongoing antibiotics for infection at another body site	-0.19	0.99	0.82	0.12	5.77	.845
Laboratory/radiological evidence of infection	1.55	0.65	4.70	1.31	16.89	.018
Perception that patient likely had another source of infection (non-LRTI)	−1.67	0.70	0.19	0.05	0.75	.018

*Note*. reference category for a prescribing decision being consistent with Pneumonia Panel results ‘yes – consistent’. LRTI = lower respiratory tract infection

All regression models significantly predicted decisions consistent with Pneumonia Panel results (Table 3). The final model (Model 4) including all predictors, significantly explained between 51 and 70% of the variance in the odds of a prescription consistent with Pneumonia Panel results (χ^2^(6) = 106.98, *p* < 0.001). Both models 3 and 4 significantly improved the overall model fit.

A prescription consistent with Pneumonia Panel results was correctly predicted by the overall model in 97% of cases, compared with only 74% of cases where prescriptions were not consistent with test results.

Receiving a positive test result (versus a negative result) increased the odds of a prescription being consistent with Pneumonia Panel results by a factor of 138.16 (Table 4). Perceiving Pneumonia Panel results to be received quickly, and the patient having confirmed laboratory and/or radiological evidence of infection increased the odds of decisions consistent with Pneumonia Panel results by factors of 12.15 and 4.70, respectively. However, if the clinician perceived the patient to likely have another (non-RTI) source of infection, the odds decreased five-fold.

**Table 4:** Overall model fit and model comparisons

Model Block	*AIC*	*BIC*	*R*^*2*^_*CS*_	*R*^*2*^_*N*_	Overall model test			*Model comparison*	*p*
					*X*^*2*^	*df*	*p*		
1	113.8	119.8	0.43	0.59	84.21	1	< .001		
2	115.8	124.8	0.43	0.59	84.21	2	< .001	1–2	.961
3	105.8	117.8	0.48	0.65	96.18	3	< .001	2–3	< .001
4	101.0	122.0	0.51	0.70	106.98	6	< .001	3–4	.013

### Stratified sensitivity analyses

Stratified logistic regression models were run restricting models to i) cases with a Positive test result, and ii) cases with a Negative test result. In the Positive test result model (*X*^*2*^(5) = 15.86, p = 0.007; Supplementary Table 4), the patient having confirmed laboratory and/or radiological evidence of infection (OR = 9.47, 95% CI [2.18, 41.13]), and the clinician not perceiving the patient to likely have another (non-RTI) source of infection (OR = 0.17, 95% CI [0.02, 0.86]) both significantly predicted prescribing decisions being consistent with Pneumonia Panel results.

In the Negative test result model (*X*^*2*^(5) = 14.76, p = 0.011; Supplementary Table 5) perceiving Pneumonia Panel results to be perceived quickly significantly predicted prescribing decisions being consistent with Pneumonia Panel results (OR = 28.05, 95% CI [2.01, 390.82]).

## Discussion

This is the first quantitative study to examine which factors are most influential in determining clinicians’ application of rapid diagnostic results to individual prescribing decisions in an intensive care setting.

We predicated the INHALE programme on the hypothesis that Pneumonia Panel results might increase clinicians’ confidence and certainty around antibiotic prescribing in relation to two key behaviours: i) avoiding an unnecessary initial antibiotic prescription and ii) changing (predominantly stopping or de-escalating) empirically-given agents early, where appropriate. However, our data suggest that the extent to which the test enables this is variable and often not enough, to enable a clinician to cross the threshold for the recommended action.

Our data inform about the conditions required for clinicians to reach their threshold for action to apply and prioritise rapid diagnostic results in their antibiotic prescribing decisions. The most influential factor increasing the likelihood of applying rapid diagnostic results was receiving a positive test result, identifying at least one pathogen. Positive results may act in a ‘confirmatory’ manner, whereas negative results may generate cognitive dissonance – namely, between what clinicians expected to find, and what the test revealed – especially when the test revealed nothing [10]. This creates a dilemma for prescribers, especially when clinicians suspect the patient may have a non-RTI infection not sought by the test, or a pneumonia due to a pathogen not represented on the test. In these cases, prescriptions were significantly less likely to be consistent with Pneumonia Panel results. Although a recent RCT showed biomarker tests to significantly reduce antibiotic duration in patients with suspected sepsis [22], another recent RCT found no relationship for suspected HAP/VAP [23]. Our findings here apply clinicians’ actual prescribing data to support previous qualitative [7, 8, 10] and vignette [24, 25] studies suggesting clinicians may be reluctant to de-escalate antibiotics for patients with suspected HAP/VAP if the patient had clinical indicators of infection, despite a negative rapid molecular diagnostic result.

We demonstrate that clinicians integrate the test result (positive vs negative) with other contextual factors when making antibiotic prescribing decisions. For example, valuing the rapidity of the Pneumonia Panel test significantly increased the likelihood of prescriptions being consistent with Pneumonia Panel results, as did the patient having additional laboratory/radiological evidence of LRTI. Furthermore, findings also suggest that clinicians’ beliefs about the test itself – particularly whether they believe the result, is likely an important factor in influencing their threshold for action. Understandably, concerns about the test, particularly the likelihood of false negative and false positive results and subsequent consequences for antibiotic under- and over-treatment, are prevalent, leading to prescribing *‘just-in-case’* of infection [7, 8, 10, 20].

**Fig. 1:**
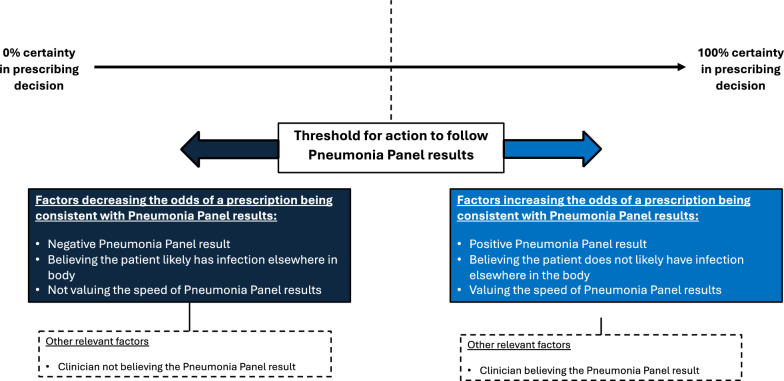
Explanatory model of factors determining a clinicians’ threshold for action to provide an initial antibiotic prescription, or to deescalate one early

Clinicians act at a point along a line of uncertainty around antibiotic prescribing. We suggest an explanatory model (Fig. 1) outlining the conditions that increase the likelihood of action – in this case – clinicians applying Pneumonia Panel results to their antibiotic prescribing decisions and prescribing in accordance with those results. Where along the line of uncertainty a clinician’s threshold for action sits will differ for each clinician and case, depending on previous experience and knowledge and individual clinical signs and symptoms.

“Correct” decisions do not automatically flow from diagnostic technology. Pneumonia Panel tests do not establish the “ground truth” of the presence of infection somewhere in the body, and therefore it would be expected that in some cases, antibiotics would be started and continued despite negative results.

Clinicians rely on more than just biomedical data and prescribing guidelines when making complex prescribing decisions. These decisions instead appear to be driven by clinicians ‘mindlines’ – “*collectively reinforced, internalised tacit guidelines*” [26, 27]. ‘Mindlines’ are flexible and iterative, shaped by clinical interactions, ‘knowledge-in-practice’ and perceptions informed by training and the experiences of themselves and others (e.g. “A colleague withheld antibiotics when needed and faced the consequences”). It would be useful for future research to examine the extent to which ‘Mindlines’ contribute to antibiotic prescribing decisions.

### Limitations

Most participants were ICU consultants (80%), and all microbiologists in the sample were from teaching or specialist hospitals in London. Further, microbiologists were relatively overrepresented as decision-makers (N = 4 contributing 14% of total responses), meaning our sample may not be representative. Although participants were recruited from a range of English ICUs, factors relevant to prescribing decision making may differ in non-ICU wards and elsewhere in the UK. Further, the COVID-19 pandemic occurred mid-way through data-collection and therefore is a potential confounder. We also acknowledge the possibility that clinicians were selective in the prescribing cases they completed questionnaires for.

We were able to make a limited assessment of the degree to which clinicians’ prescribing behaviour was consistent with Pneumonia Panel results. Two aspects of clinician prescribing behaviour are of interest here: i) whether prescriptions were consistent with Pneumonia Panel results which can inform a yes/no decision about whether an antibiotic prescription is warranted, and ii) whether prescriptions were consistent with further guidance (the algorithm) advising on the specific antibiotic that could be used to combat the specific pathogen(s) reported by the test. In the present study, we observed that adherence to the INHALE trial prescribing algorithm was low, and that there was a discrepancy between clinicians’ perceptions of their prescribing and their objective adherence to the algorithm. However, as this was outside the scope of the present research, we have been unable to understand reasons for this. Our findings highlight a number of factors influencing antibiotic prescribing decision-making within the context of the availability of rapid diagnostic results. However, the presented analyses do not allow for an exploration of the mechanisms underlying these relationships. This would be important to address in future research. Additionally, more evidence is warranted regarding the safety of withholding or narrowing antibiotic therapy based on negative rapid diagnostic results. This should be considered when interpreting patterns of prescribing ‘consistency’ in the presented analyses.

Further, our stratified sensitivity analyses necessarily have smaller sample sizes and as such findings should be interpreted cautiously. Nonetheless, the overall pattern from these results are consistent with the main model.

Finally, we could not analyse the influence, contribution and “value” to evidence-based prescribing of the rapid antibiotic resistance detection provided by this platform in view of the very low rates of antimicrobial resistance in the UK when compared to other countries.

### Study implications

Findings highlight the complexities of ICU antibiotic decision making and highlight the challenges of successfully implementing use of rapid microbiological diagnostics. Negative results present a particular challenge, as they can conflict with the perceptions and beliefs that contribute to antibiotic decision-making. Clinicians have a strong imperative to protect the patient and themselves, and therefore under circumstances of uncertainty when the clinical presentation of the patient conflicts with a negative result, it often leads to antibiotic prescribing *‘just-in-case’* of infection. In some cases, this is likely ‘right’ and in others (with no pathogen present) ‘wrong’.

This reluctance to rely on new technologies to provide targeted therapy faster, or to change (preferentially stop or de-escalate) antibiotics early if appropriate, suggests that deployment of the technology of itself, may not be sufficient for widespread implementation. Rather, we suggest that diagnostic technologies, however high-performance, will only deliver the promise of better healthcare when combined with context specific implementation strategies. They must be behaviourally intelligent, connecting with how clinicians think and behave, recognising the human elements underpinning antibiotic choice and prescription.

## Conclusion

Rapid diagnostics such as the Pneumonia Panel may increase certainty around antibiotic prescribing in relation to two key aspects: i) avoid the initial prescribing of unnecessary antibiotics and ii) to stop or de-escalate a prescription early, where appropriate. However, we suggest that the extent to which such tests do this is variable and often insufficient on its own to enable the recommended action. The most influential factor determining antibiotic prescribing decisions was the test result itself. This creates tension when the test result conflicts with the clinical presentation of the patient, juxtaposing the art and science of medicine. Although many clinicians were willing to apply positive rapid diagnostic results to their prescribing, when results indicated the absence of infection, many were reluctant to withhold antibiotics or to de-escalate early where appropriate, ‘*erring on the side of caution*’.

We also identify that other contextual factors such as clinicians’ perceptions about the speed of the test and the condition of the patient are likely important in influencing a clinicians’ threshold to act. Findings have implications for the implementation of rapid diagnostics, particularly for the management of negative results in practice. Simply having the technology available may not deliver the desired impact on stewardship, usage and cost-effectiveness.

## Supplementary information

Additional file1 (DOCX 16 KB)

Additional file2 (DOCX 16 KB)

Additional file3 (DOCX 26 KB)

Additional file4 (DOCX 15 KB)

Additional file5 (DOCX 15 KB)

## Data Availability

No data are available for this study due to compromising anonymity.

## List of abbreviations

AMR: Antimicrobial resistance
EMA: Ecological momentary assessment
EVP: Events-per-variable
HAP/VAP: Hospital-acquired and ventilator-associated pneumonia
ICU: Intensive care unit
RCT: Randomised-control trial
RTI: Respiratory tract infection
SOP: Standard operating procedure
UK: United Kingdom

## Footnotes

1 One hospital participating in the INHALE RCT did not start recruiting to the trial until after sampling for this study had concluded.

2 Activity defined as likely susceptibility to the antibiotic given, based on laboratory results or epidemiological data if the pathogen was not grown in the laboratory

3 Of the included 159 intervention-arm questionnaires, 123 were received before the pause due to the COVID-19 pandemic; 36 were received after recruitment re-started.
