# Supplementary material for: Machine says go, doctor says no: an ecological momentary assessment analysis examining clinicians’ perceptions of, and their antibiotic prescribing behaviour when using rapid molecular diagnostic tests in intensive care
Source: Antimicrob Resist Infect Control. 2026 Mar 24;15:42. doi: 10.1186/s13756-025-01690-8 (PMC13023110; doi:10.1186/s13756-025-01690-8)
Supplement: Supplementary file 1 — Additional file1 (DOCX 16 KB) [file 13756_2025_1690_MOESM1_ESM.docx]

**Supplementary Material 1**

*Organisms detected by the BioFire FilmArray Pneumonia Panel*

| **Bacteria (semi-quantitative)** | **Viruses** |
| --- | --- |
| *Acinetobacter calcoaceticus-baumannii* complex  *Enterobacter cloacae* complex  *Escherichia coli*  *Haemophilus influenzae*  *Klebsiella aerogenes*  *Klebsiella oxytoca*  *Klebsiella pneumoniae* group  *Moraxella catarrhalis*  *Proteus* spp.  *Pseudomonas aeruginosa*  *Serratia marcescens*  *Staphylococcus aureus*  *Streptococcus agalactiae*  *Streptococcus pneumoniae*  *Streptococcus pyogenes* | Adenovirus  Coronavirus (NOT SARS-CoV-2)  Human Metapneumovirus  Human Rhinovirus/Enterovirus  Influenza A  Influenza B  Parainfluenza Virus  Respiratory Syncytial Virus |
| **Atypical bacteria (qualitative)** | **Antibiotic resistance genes** |
| *Chlamydia pneumoniae*  *Legionella pneumophila*  *Mycoplasma pneumoniae* | Methicillin resistance:   - *mec*A/C and MREJ   Carbapenemases:   - KPC - NDM - Oxa-48-like - VIM - IMP   ESBL:   - CTX-M |
