# Supplementary material for: Machine says go, doctor says no: an ecological momentary assessment analysis examining clinicians’ perceptions of, and their antibiotic prescribing behaviour when using rapid molecular diagnostic tests in intensive care
Source: Antimicrob Resist Infect Control. 2026 Mar 24;15:42. doi: 10.1186/s13756-025-01690-8 (PMC13023110; doi:10.1186/s13756-025-01690-8)
Supplement: Supplementary file 2 — Additional file2 (DOCX 16 KB) [file 13756_2025_1690_MOESM2_ESM.docx]

**Supplementary Material 2**

*Participating hospital characteristics*

| **Hospital number** | **Location in the UK** | **Hospital type** | **Hospital bed number** | **ICU bed number** | **Number of questionnaires completed** | | |
| --- | --- | --- | --- | --- | --- | --- | --- |
|  |  |  |  |  | **Intervention** | **Control** | **Total** |
| 1 | Liverpool | Teaching | 810 | 23 | 13 | 12 | 25 |
| 2 | London | Teaching | 430 | 11 | 24 | 17 | 41 |
| 3 | London | Private | 118 | 7 | 5 | 7 | 12 |
| 4 | West Midlands | General | 596 | 25 | 4 | 0 | 4 |
| 5 | London | Specialist paediatric | 425 | 27 | 18 | 19 | 37 |
| 6 | Hertfordshire | District general | 663 | 19 | 7 | 5 | 12 |
| 7 | Norfolk | Teaching | 520 | 12 | 1 | 1 | 2 |
| 8 | Liverpool | Teaching | 710 | 32 | 12 | 10 | 22 |
| 9 | Birmingham | Specialist paediatric | 300 | 31 | 20 | 11 | 31 |
| 10 | London | Teaching | 830 | 46 | 11 | 8 | 19 |
| 11 | Staffordshire | Teaching | 1,328 | 12 | 15 | 18 | 33 |
| 12 | London | Teaching | 720 | 35 | 32 | 23 | 55 |
| 13 | London | Specialist paediatric heart and lung | 295 | 16 | 2 | 0 | 2 |

*Paediatric clinicians participated in Hospitals 5, 9, and 13; clinicians at remaining sites treated adult patients
