# Supplementary material for: Machine says go, doctor says no: an ecological momentary assessment analysis examining clinicians’ perceptions of, and their antibiotic prescribing behaviour when using rapid molecular diagnostic tests in intensive care
Source: Antimicrob Resist Infect Control. 2026 Mar 24;15:42. doi: 10.1186/s13756-025-01690-8 (PMC13023110; doi:10.1186/s13756-025-01690-8)
Supplement: Supplementary file 3 — Additional file3 (DOCX 26 KB) [file 13756_2025_1690_MOESM3_ESM.docx]

**Supplementary Material 3**

Questionnaire administration flow diagram
