# Supplementary material for: Machine says go, doctor says no: an ecological momentary assessment analysis examining clinicians’ perceptions of, and their antibiotic prescribing behaviour when using rapid molecular diagnostic tests in intensive care
Source: Antimicrob Resist Infect Control. 2026 Mar 24;15:42. doi: 10.1186/s13756-025-01690-8 (PMC13023110; doi:10.1186/s13756-025-01690-8)
Supplement: Supplementary file 4 — Additional file4 (DOCX 15 KB) [file 13756_2025_1690_MOESM4_ESM.docx]

**Supplementary Material 4**

*Sensitivity analyses stratified by Pneumonia Panel result (Positive results only)*

|  |  |  |  | *95% Confidence Interval* | |  |
| --- | --- | --- | --- | --- | --- | --- |
|  | *B* | *SE* | *ExpB* | *Lower* | *Upper* | *p* |
| Intercept | 1.28 | 1.13 | 3.59 | 0.39 | 32.66 | .257 |
| Believing Pneumonia Panel results | -0.58 | 1.17 | 0.56 | 0.06 | 5.60 | .623 |
| Influenced by quick speed of Pneumonia Panel | 1.24 | 1.12 | 3.45 | 0.39 | 30.92 | .268 |
| Ongoing antibiotics for infection at another body site | -0.12 | 1.08 | 0.89 | 0.11 | 7.37 | .913 |
| Laboratory/radiological evidence of infection | 2.25 | 0.75 | 9.47 | 2.18 | 41.13 | .003 |
| Perception that patient likely had another source of infection (non-LRTI) | -1.76 | 0.82 | 0.17 | 0.02 | 0.86 | .033 |
