# Supplementary material for: Machine says go, doctor says no: an ecological momentary assessment analysis examining clinicians’ perceptions of, and their antibiotic prescribing behaviour when using rapid molecular diagnostic tests in intensive care
Source: Antimicrob Resist Infect Control. 2026 Mar 24;15:42. doi: 10.1186/s13756-025-01690-8 (PMC13023110; doi:10.1186/s13756-025-01690-8)
Supplement: Supplementary file 5 — Additional file5 (DOCX 15 KB) [file 13756_2025_1690_MOESM5_ESM.docx]

**Supplementary Material 5**

*Sensitivity analyses stratified by Pneumonia Panel result (Negative results only)**

|  |  |  |  | *95% Confidence Interval* | |  |
| --- | --- | --- | --- | --- | --- | --- |
|  | *B* | *SE* | *ExpB* | *Lower* | *Upper* | *p* |
| Intercept | -2.88 | 1.90 | 0.06 | 0.00 | 2.30 | .128 |
| Believing Pneumonia Panel results | 0.73 | 1.49 | 2.08 | 0.11 | 38.48 | .624 |
| Influenced by quick speed of Pneumonia Panel | 3.33 | 1.34 | 28.05 | 2.01 | 390.82 | .013 |
| Ongoing antibiotics for infection at another body site* | -15.50 | 5960.65 | 1.849e-7 | 0.00 | Inf | .998 |
| Laboratory/radiological evidence of infection | -0.92 | 1.48 | 0.40 | 0.02 | 7.24 | .535 |
| Perception that patient likely had another source of infection (non-LRTI)* | -18.19 | 3934.43 | 1.263e-8 | 0.00 | Inf | .996 |

*Results reflect quasi-complete separation caused by very small cell counts; estimates are not statistically interpretable.
